# Supplementary material for: Systematic evaluation of lecithin:cholesterol acyltransferase binding sites in apolipoproteins via peptide based nanodiscs: regulatory role of charged residues at positions 4 and 7
Source: PLoS Comput Biol. 2024 May 28;20(5):e1012137. doi: 10.1371/journal.pcbi.1012137 (PMC11161081; doi:10.1371/journal.pcbi.1012137)

**SUPPORTING INFORMATION**

**Systematic evaluation of lecithin:cholesterol acyltransferase binding sites in apolipoproteins via peptide based nanodiscs: regulatory role of charged residues at positions 4 and 7**

Akseli Niemelä, Artturi Koivuniemi

Division of Pharmaceutical Biosciences, Faculty of Pharmacy, University of Helsinki, Helsinki, Finland

**Table A.** Peptide sequences in simulated systems.

**Table B.** Hydrogen bonding results of atomistic systems. Peptide residues 4 and 7 and LCAT residues 53, 236 and 238 are bolded. Bonds with above 40 % occupancy are shown.

**Table C.** Occupancy results of 22A D4 mutation screen.

**Table D.** All 22 residue long sequences with DE4-QR7 moieties in a variety of proteins.

**Fig A.** LCAT binding pose from different perspectives on HDL particle. Snapshot of the last frame of helix 7 simulation. LCAT is coloured according to Fig 1 and apoA1 helixes according to Fig 4. Note the different position the peptide takes compared to Fig 1B.

**Fig B.** The helix of apoA1 LCAT is bound to as a function of time. Simulations were started at helixes 1, 2, 4, 5, 6, 7, 8 and 10, but 2, 5, 8 and 10 were cancelled. As these helixes utilized a different binding orientation than 22 amino acid long peptides (Fig 1 vs Fig A) the occupancy condition was adjusted to any helix whose backbone bead distances between helix residue 2 and LCAT Q229 and helix residue 20 and LCAT L239 is within 1.2 nm.

**Fig C.** Angle-distance profiles of CG systems with unnormalized colorbars. The apoA1 helix mix system’s bin counts are not comparable to other systems as it had 14 x 14 peptide pairs, unlike the other systems with 378 peptide pairs.

**Fig D.** LCAT position density plots of CG systems. X-axis is distance from 0 to 10 nm perpendicular to nanodisc normal and Y-axis is distance from -5 to 5 nm parallel to nanodisc normal. A plane was fitted to all DMPC beads and LCAT’s position relative to it was determined with the same method as described in reference [1]. An illustrative superimposed image is included. The colorbar is normalized by the maximum bin count of apoE helix 1. Systems with less than 10 % peptide dissociation are marked with a dashed red border.

**Fig E.** Peptide position density plots of CG systems. X-axis is distance from 0 to 7 nm perpendicular to nanodisc normal and Y-axis is distance from 0 to 3.5 nm parallel to nanodisc normal. A plane was fitted to all DMPC beads and peptides’ positions relative to it were determined with a script. An illustrative superimposed image is included. The colorbar is normalized by the maximum bin count of 22A-R7L. Systems with less than 10 % peptide dissociation are marked with a dashed red border.

**Fig F.** Correlation between simulation results for all CG systems with single helical peptides. Variable “pep_pos_maxval” is the maximum value in a system’s peptide position density plot, “lcat_pos_maxval” in LCAT position plot and “angle_distance_maxval” in angle-distance plots. Correlation matrixes were generated with GGally [2,3]. *p<0.05, **p<0.01, ***p<0.001. Evidently in systems with less peptides on the nanodisc LCAT’s movement was more restricted, likely due to entropic forces driving LCAT onto the lipid bilayer. With a peptide rich surface, LCAT is able to sample different angles of the nanodisc perimeter more effectively.

**Fig G.** Correlation between dissociation and hydrophilicity or hydrophobic moment for non-22A based helixes. Cancelled systems were included with 100 % dissociation.

1. Giorgi L, Niemelä A, Kumpula E-P, Natri O, Parkkila P, Huiskonen JT, et al. Mechanistic Insights into the Activation of Lecithin–Cholesterol Acyltransferase in Therapeutic Nanodiscs Composed of Apolipoprotein A-I Mimetic Peptides and Phospholipids. Mol Pharmaceutics. 2022:19: 4135-4148.

2. Schloerke B, Cook D, Larmarange J, Briatte F, Marbach M, Thoen E, Elberg A, Crowley J (2024). GGally: Extension to 'ggplot2'. R package version 2.2.1, https://github.com/ggobi/ggally, https://ggobi.github.io/ggally/.

3. R Core Team (2023). R: A Language and Environment for Statistical Computing. R Foundation for Statistical Computing, Vienna, Austria. https://R-project.org/.

**Table A**

| ID | peptide | peptide sequence |
| --- | --- | --- |
| F1 | apoA1 helix 1 | LKLLDNWDSVTSTFSKLREQLG |
| F2 | apoA1 helix 2 | PVTQEFWDNLEKETEGLRQEMS |
| F3 | apoA1 helix 4 | PYLDDFQKKWQEEMELYRQKVE |
| F4 | apoA1 helix 5 | PLRAELQEGARQKLHELQEKLS |
| F5 | apoA1 helix 6 | PLGEEMRDRARAHVDALRTHLA |
| F6 | apoA1 helix 7 | PYSDELRQRLAARLEALKENGG |
| F7 | apoA1 helix 8 | ARLAEYHAKATEHLSTLSEKAK |
| F8 | apoA1 helix 10 | PVLESFKVSFLSALEEYTKKLNTQ |
| E1 | apoE helix 1 | ALMDETMKELKAYKSELEEQLT |
| E2 | apoE helix 2 | PVAEETRARLSKELQAAQARLG |
| E3 | apoE helix 3 | ADMEDVCGRLVQYRGEVQAMLG |
| E4 | apoE helix 4 | QSTEELRVRLASHLRKLRKRLL |
| E5 | apoE helix 5 | RDADDLQKRLAVYQAGAREGAE |
| E6 | apoE helix 6 | RGLSAIRERLGPLVEQGRVRAA |
| E7 | apoE helix 7 | TVGSLAGQPLQERAQAWGERLR |
| E8 | apoE helix 8 | ARMEEMGSRTRDRLDEVKEQVA |
| L1 | 22A | PVLDLFRELLNELLEALKQKLK |
| L2 | 22A-R7Q | PVLDLFQELLNELLEALKQKLK |
| R1 | 22A-R7D | PVLDLFDELLNELLEALKQKLK |
| R2 | 22A-R7H | PVLDLFHELLNELLEALKQKLK |
| R3 | 22A-R7C | PVLDLFCELLNELLEALKQKLK |
| R4 | 22A-R7L | PVLDLFLELLNELLEALKQKLK |
| R5 | 22A-R7K | PVLDLFKELLNELLEALKQKLK |
| D1 | 22A-D4E | PVLELFRELLNELLEALKQKLK |
| D2 | 22A-D4K | PVLKLFRELLNELLEALKQKLK |
| D3 | 22A-D4L | PVLLLFRELLNELLEALKQKLK |
| D4 | 22A-D4N | PVLNLFRELLNELLEALKQKLK |
| D5 | 22A-D4N-R7Q | PVLNLFQELLNELLEALKQKLK |
| N1 | apoA1 helix B | KETEGLRQEMSKDLEEVKAKVQ |
| N2 | apoA1 helix F | ELQEGARQKLHELQEKLSPLGE |
| N3 | apoA1 helix H | EMRDRARAHVDALRTHLAPYSD |
| N4-5 | apoA1 helix I | AHVDALRTHLAPYSDELRQRLA |
| N6 | apoE helix B | RFWDYLRWVQTLSEQVQEELLS |
| N7 | apoE helix C | TLSEQVQEELLSSQVTQELRAL |
| N8 | apoE helix F | RDADDLQKRLAVYQAGAREGAE |
| N9 | apoE helix H | PLQERAQAWGERLRARMEEMGS |
| N10 | apoE helix I | AWGERLRARMEEMGSRTRDRLD |
| N11 | apoE helix J | KLEEQAQQIRLQAEAFQARLKS |
| N12 | apoE helix L | PLVEDMQRQWAGLVEKVQAAVG |
| N13 | apoB100 helix A | CPKDATRFKHLRKYTYNYEAES |
| N14 | apoB100 helix M | ATYELQREDRALVDTLKFVTQA |
| N15 | apoB100 helix O | ADYESLRFFSLLSGSLNSHGLE |
| N16 | apoB100 helix X | KLKETIQKLSNVLQQVKIKDYF |
| N17 | apoB100 helix Z | ETNDKIREVTQRLNGEIQALEL |
| N18 | apoB100 helix AA | KIREVTQRLNGEIQALELPQKA |
| N19 | apoB100 helix AB | QALELPQKAEALKLFLEETKAT |
| N20 | apoB100 helix AD | ETLEDTRDRMYQMDIQQELQRY |
| N21 | apoB100 helix AE | YQMDIQQELQRYLSLVGQVYST |
| N22 | apoB100 helix AF | IQQELQRYLSLVGQVYSTLVTY |
| N23 | apoB100 helix AG | RHFEKNRNNALDFVTKSYNETK |
| N24 | apoB100 helix AN | KTTEVLRNLQDLLQFIFQLIED |
| N25 | apoB100 helix AO | NLQDLLQFIFQLIEDNIKQLKE |
| N26 | apoB100 helix AP | KFNEFIQNELQEASQELQQIHQ |
| N27 | apoB100 helix AQ | ELQEASQELQQIHQYIMALREE |
| N28 | apoB100 helix AR | ASQELQQIHQYIMALREEYFDP |
| N29 | apoB100 helix AS | IISDYHQQFRYKLQDFSDQLSD |
| N30 | apoC1 helix A | TLEDKARELISRIKQSELSAKM |
| N31 | apoL1 helix B | NEADELRKALDNLARQMIMKDK |
| N32 | apoL1 helix D | FLKEFPRLKSELEDNIRRLRAL |
| N33 | apoL1 helix E | ELEDNIRRLRALADGVQKVHKG |
| N34 | LCAT helix D | QQEEYYRKLAGLVEEMHAAYGK |
| N35 | serum amyloid A1/2 helix B | GARDMWRAYSDMREANYIGSDK |
| N36 | serum amyloid A4 helix B | GVGDMGRAYWDIMISNHQNSNR |
| N37 | albumin helix C | AFTECCQAADKAACLLPKLDEL |
| N38 | albumin helix D | PKLDELRDEGKASSAKQRLKCA |
| N39 | albumin helix G | TLVEVSRNLGKVGSKCCKHPEA |
| N40 | albumin helix I | TLSEKERQIKKQTALVELVKHK |

**Table B**

| atomistic simulation system | peptide residue | LCAT residue | occupancy (%) |
| --- | --- | --- | --- |
| 22A 1 | **ARG7-Side** | **ASP73-Side** | 98.1 |
| 22A 2 | **ARG7-Side** | **ASP73-Side** | 99.9 |
|  | GLU8-Side | **LYS238-Side** | 81.4 |
|  | **ASP4-Side** | **SER236-Side** | 74.9 |
| 22A 3 | **ARG7-Side** | **ASP73-Side** | 89.0 |
| 22A-R7Q 1 | **ASP4-Side** | **SER236-Side** | 90.2 |
|  | PRO1-Main | ASP335-Side | 40.9 |
| 22A-R7Q 2 | PRO1-Main | ASP227-Side | 49.4 |
|  | **ASP4-Side** | **SER236-Side** | 45.1 |
| 22A-R7Q 3 | **ASP4-Side** | **LYS238-Side** | 66.2 |
| apoA1 helix 4 | ARG18-Side | ASP44-Side | 98.7 |
|  | **ASP4-Side** | **LYS238-Side** | 60.4 |
| apoA1 helix 6 | **ARG7-Side** | ASP227-Side | 99.7 |
|  | **ARG7-Side** | **ASP73-Side** | 99.5 |
|  | **GLU4-Side** | **LYS238-Side** | 64.3 |
| apoA1 helix 7 | **ARG7-Side** | **ASP73-Side** | 95.5 |
|  | **ASP4-Side** | **SER236-Side** | 64.4 |
|  | SER3-Side | ASP227-Side | 43.9 |
| apoE helix 1 1 | LYS11-Side | **ASP73-Side** | 64.5 |
|  | LYS11-Side | GLU242-Side | 43.2 |
| apoE helix 1 2 | **ASP4-Side** | ARG244-Side | 43.6 |
|  | ALA1-Main | SER225-Main | 41.1 |
| apoB100 helix AS | ILE1-Main | ASP227-Side | 96.7 |
|  | ARG10-Side | **ASP73-Side** | 95.3 |
|  | **ASP4-Side** | **SER236-Side** | 92.1 |
|  | SER3-Side | ASP227-Side | 82.9 |
|  | SER3-Main | ASP227-Side | 82.4 |
|  | ILE2-Main | ASP227-Side | 54.9 |
|  | TYR11-Side | LYS240-Main | 48.8 |
|  | **ASP4-Side** | ILE237-Main | 44.5 |

**Table C**

| peptide | occupancy (%) |
| --- | --- |
| 22A-D4E | 10.2±2.4 |
| 22A-D4K | 15.2±1.2 |
| 22A-D4L | 21.1±5.4 |
| 22A-D4N | 5.6±0.4 |
| 22A-D4N-R7Q | 2.8±1.3 |

**Table D**

| protein | ID | hydro-philicity | helix  penalty  (kJ/mol) | resid  start | resid  end | sequence |
| --- | --- | --- | --- | --- | --- | --- |
| apoA1 | A | 0.255 | 2.012 | 21 | 42 | VLKDSGRDYVSQFEGSALGKQL |
| apoA1 | B | 1.132 | 1.692 | 77 | 98 | KETEGLRQEMSKDLEEVKAKVQ |
| apoA1 | C | 0.623 | 1.693 | 99 | 120 | PYLDDFQKKWQEEMELYRQKVE |
| apoA1 | D | 0.859 | 1.96 | 110 | 131 | EEMELYRQKVEPLRAELQEGAR |
| apoA1 | E | 0.664 | 1.978 | 117 | 138 | QKVEPLRAELQEGARQKLHELQ |
| apoA1 | F | 0.677 | 2.155 | 125 | 146 | ELQEGARQKLHELQEKLSPLGE |
| apoA1 | G | 0.564 | 1.47 | 143 | 164 | PLGEEMRDRARAHVDALRTHLA |
| apoA1 | H | 0.555 | 2.042 | 147 | 168 | EMRDRARAHVDALRTHLAPYSD |
| apoA1 | I | 0.186 | 2.056 | 154 | 175 | AHVDALRTHLAPYSDELRQRLA |
| apoA1 | J | 0.623 | 1.532 | 165 | 186 | PYSDELRQRLAARLEALKENGG |
| apoA1 | K | 0.6 | 1.426 | 166 | 187 | YSDELRQRLAARLEALKENGGA |
| apoA1 | L | -0.159 | 2.205 | 209 | 230 | PALEDLRQGLLPVLESFKVSFL |
| apoA1 | M | -0.145 | 2.305 | 210 | 231 | ALEDLRQGLLPVLESFKVSFLS |
| apoE | A | 0.273 | 2.292 | 10 | 31 | PEPELRQQTEWQSGQRWELALG |
| apoE | B | -0.136 | 1.8 | 32 | 53 | RFWDYLRWVQTLSEQVQEELLS |
| apoE | C | 0.155 | 1.553 | 42 | 63 | TLSEQVQEELLSSQVTQELRAL |
| apoE | D | 0.541 | 1.249 | 84 | 105 | PVAEETRARLSKELQAAQARLG |
| apoE | E | 0.641 | 1.297 | 128 | 149 | QSTEELRVRLASHLRKLRKRLL |
| apoE | F | 0.795 | 1.572 | 150 | 171 | RDADDLQKRLAVYQAGAREGAE |
| apoE | G | -0.086 | 2.344 | 183 | 204 | PLVEQGRVRAATVGSLAGQPLQ |
| apoE | H | 0.618 | 1.415 | 202 | 223 | PLQERAQAWGERLRARMEEMGS |
| apoE | I | 1.014 | 1.671 | 209 | 230 | AWGERLRARMEEMGSRTRDRLD |
| apoE | J | 0.482 | 1.177 | 242 | 263 | KLEEQAQQIRLQAEAFQARLKS |
| apoE | K | 0.036 | 1.998 | 252 | 273 | LQAEAFQARLKSWFEPLVEDMQ |
| apoE | L | -0.009 | 1.736 | 267 | 288 | PLVEDMQRQWAGLVEKVQAAVG |
| apoE | M | -0.027 | 1.826 | 268 | 289 | LVEDMQRQWAGLVEKVQAAVGT |
| apoE | N | -0.032 | 2.914 | 278 | 299 | GLVEKVQAAVGTSAAPVPSDNH |
| LCAT | A | -0.236 | 2.759 | 74 | 95 | CWIDNTRVVYNRSSGLVSNAPG |
| LCAT | B | 0.705 | 2.83 | 134 | 155 | VRDETVRAAPYDWRLEPGQQEE |
| LCAT | C | 0.35 | 2.222 | 146 | 167 | WRLEPGQQEEYYRKLAGLVEEM |
| LCAT | D | 0.277 | 1.631 | 152 | 173 | QQEEYYRKLAGLVEEMHAAYGK |
| LCAT | E | 0.073 | 2.747 | 238 | 259 | KLKEEQRITTTSPWMFPSRMAW |
| LCAT | F | -0.327 | 1.978 | 274 | 295 | TGRDFQRFFADLHFEEGWYMWL |
| apoB100 | A | 0.455 | 2.246 | 12 | 33 | CPKDATRFKHLRKYTYNYEAES |
| apoB100 | B | -0.059 | 2.339 | 53 | 74 | VELEVPQLCSFILKTSQCTLKE |
| apoB100 | C | 0.077 | 3.594 | 107 | 128 | AIPEGKQVFLYPEKDEPTYILN |
| apoB100 | D | -0.064 | 2.153 | 142 | 163 | ETEEAKQVLFLDTVYGNCSTHF |
| apoB100 | E | 0.432 | 2.989 | 178 | 199 | TERDLGQCDRFKPIRTGISPLA |
| apoB100 | F | 0.173 | 1.519 | 220 | 241 | YTLDAKRKHVAEAICKEQHLFL |
| apoB100 | G | 0.109 | 2.053 | 442 | 463 | YLMEQIQDDCTGDEDYTYLILR |
| apoB100 | H | 0.791 | 3.07 | 508 | 529 | EPKDKDQEVLLQTFLDDASPGD |
| apoB100 | I | -0.141 | 2.076 | 785 | 806 | MIGEVIRKGSKNDFFLHYIFME |
| apoB100 | J | -0.314 | 2.691 | 863 | 884 | IIPDFARSGVQMNTNFFHESGL |
| apoB100 | K | -0.373 | 2.606 | 928 | 949 | PLIENRQSWSVCKQVFPGLNYC |
| apoB100 | L | 0.477 | 1.812 | 980 | 1001 | PTGEIEQYSVSATYELQREDRA |
| apoB100 | M | 0.25 | 1.701 | 991 | 1012 | ATYELQREDRALVDTLKFVTQA |
| apoB100 | N | -0.2 | 2.968 | 1284 | 1305 | KMLETVRTPALHFKSVGFHLPS |
| apoB100 | O | -0.164 | 2.016 | 1577 | 1598 | ADYESLRFFSLLSGSLNSHGLE |
| apoB100 | P | 0.45 | 2.07 | 1961 | 1982 | NNNEYSQDLDAYNTKDKIGVEL |
| apoB100 | Q | 0.436 | 2.403 | 2017 | 2038 | DAVEKPQEFTIVAFVKYDKNQD |
| apoB100 | R | 0.114 | 2.542 | 2031 | 2052 | VKYDKNQDVHSINLPFFETLQE |
| apoB100 | S | -0.1 | 1.869 | 2045 | 2066 | PFFETLQEYFERNRQTIIVVLE |
| apoB100 | T | 0.232 | 1.837 | 2052 | 2073 | EYFERNRQTIIVVLENVQRNLK |
| apoB100 | U | 0.023 | 1.895 | 2063 | 2084 | VVLENVQRNLKHINIDQFVRKY |
| apoB100 | V | 0.105 | 1.577 | 2234 | 2255 | QIQEKLQQLKRHIQNIDIQHLA |
| apoB100 | W | 0.25 | 1.685 | 2319 | 2340 | ERYEVDQQIQVLMDKLVELAHQ |
| apoB100 | X | 0.223 | 1.764 | 2342 | 2363 | KLKETIQKLSNVLQQVKIKDYF |
| apoB100 | Y | 0.659 | 2.122 | 2401 | 2422 | KSFDYHQFVDETNDKIREVTQR |
| apoB100 | Z | 0.591 | 1.799 | 2411 | 2432 | ETNDKIREVTQRLNGEIQALEL |
| apoB100 | AA | 0.45 | 2.108 | 2415 | 2436 | KIREVTQRLNGEIQALELPQKA |
| apoB100 | AB | 0.323 | 1.761 | 2428 | 2449 | QALELPQKAEALKLFLEETKAT |
| apoB100 | AC | -0.455 | 1.654 | 2452 | 2473 | VYLESLQDTKITLIINWLQEAL |
| apoB100 | AD | 0.655 | 1.743 | 2487 | 2508 | ETLEDTRDRMYQMDIQQELQRY |
| apoB100 | AE | -0.373 | 1.886 | 2497 | 2518 | YQMDIQQELQRYLSLVGQVYST |
| apoB100 | AF | -0.627 | 1.942 | 2501 | 2522 | IQQELQRYLSLVGQVYSTLVTY |
| apoB100 | AG | 0.577 | 1.98 | 3179 | 3200 | RHFEKNRNNALDFVTKSYNETK |
| apoB100 | AH | -0.136 | 3.84 | 3211 | 3232 | SHDELPRTFQIPGYTVPVVNVE |
| apoB100 | AI | 0.318 | 1.793 | 3345 | 3366 | SVIDALQYKLEGTTRLTRKRGL |
| apoB100 | AJ | 0.218 | 2.359 | 3585 | 3606 | DFPDLGQEVALNANTKNQKIRW |
| apoB100 | AK | 0.164 | 2.327 | 3935 | 3956 | GKYEGLQEWEGKAHLNIKSPAF |
| apoB100 | AL | 0.586 | 1.84 | 4020 | 4041 | ESDEETQIKVNWEEEAASGLLT |
| apoB100 | AM | 0.195 | 1.707 | 4116 | 4137 | EWKDKAQNLYQELLTQEGQASF |
| apoB100 | AN | -0.05 | 1.811 | 4264 | 4285 | KTTEVLRNLQDLLQFIFQLIED |
| apoB100 | AO | -0.009 | 1.649 | 4271 | 4292 | NLQDLLQFIFQLIEDNIKQLKE |
| apoB100 | AP | 0.168 | 1.727 | 4331 | 4352 | KFNEFIQNELQEASQELQQIHQ |
| apoB100 | AQ | 0.236 | 1.412 | 4339 | 4360 | ELQEASQELQQIHQYIMALREE |
| apoB100 | AR | -0.045 | 2.193 | 4343 | 4364 | ASQELQQIHQYIMALREEYFDP |
| apoB100 | AS | 0.109 | 1.967 | 4460 | 4481 | IISDYHQQFRYKLQDFSDQLSD |
| apoB100 | AT | -0.482 | 1.742 | 4486 | 4507 | FIAESKRLIDLSIQNYHTFLIY |
| apoD | A | 0.105 | 2.002 | 34 | 55 | TTFENGRCIQANYSLMENGKIK |
| apoF | A | -0.077 | 2.662 | 7 | 28 | CENEKEQAVHNVVQLLPGVGTF |
| apoF | B | 0.455 | 1.755 | 45 | 66 | KARERGRDGAIDLGYDLLMTMA |
| apoF | C | 0.45 | 1.86 | 109 | 130 | TTKEGLRAISDVSDLEETTTLA |
| apoH | A | 0.355 | 2.678 | 289 | 310 | SYTEDAQCIDGTIEVPKCFKEH |
| apoL1 | A | 0.782 | 1.748 | 72 | 93 | AAAELPRNEADELRKALDNLAR |
| apoL1 | B | 0.877 | 1.321 | 79 | 100 | NEADELRKALDNLARQMIMKDK |
| apoL1 | C | 0.177 | 2.391 | 101 | 122 | NWHDKGQQYRNWFLKEFPRLKS |
| apoL1 | D | 0.645 | 1.905 | 113 | 134 | FLKEFPRLKSELEDNIRRLRAL |
| apoL1 | E | 0.714 | 1.761 | 123 | 144 | ELEDNIRRLRALADGVQKVHKG |
| apoL1 | F | -0.109 | 2.239 | 133 | 154 | ALADGVQKVHKGTTIANVVSGS |
| apoL1 | G | -0.341 | 2.426 | 298 | 319 | SILEMSRGVKLTDVAPVSFFLV |
| apoM | A | 0.173 | 3.338 | 22 | 43 | QCPEHSQLTTLGVDGKEFPEVH |
| apoM | B | -0.209 | 3.661 | 133 | 154 | MLNETGQGYQRFLLYNRSPHPP |
| apo(A) | A | 0.032 | 2.429 | 1365 | 1386 | GVQDCYRGDGQSYRGTLSTTIT |
| apo(A) | B | -0.005 | 3.051 | 1726 | 1747 | AAQEPHRHSTFIPGTNKWAGLE |
| apo(A) | C | 0.032 | 2.01 | 1870 | 1891 | QEIEVSRLFLEPTQADIALLKL |
| apo(A) | D | -0.291 | 2.57 | 1877 | 1898 | LFLEPTQADIALLKLSRPAVIT |
| apo(A) | E | 0.514 | 3.078 | 1962 | 1983 | RGTDSCQGDSGGPLVCFEKDKY |
| albumin | A | 0.768 | 3.54 | 92 | 113 | AKQEPERNECFLQHKDDNPNLP |
| albumin | B | -0.495 | 2.523 | 138 | 159 | YLYEIARRHPYFYAPELLFFAK |
| albumin | C | 0.118 | 2.059 | 164 | 185 | AFTECCQAADKAACLLPKLDEL |
| albumin | D | 1.041 | 1.463 | 180 | 201 | PKLDELRDEGKASSAKQRLKCA |
| albumin | E | -0.209 | 2.015 | 330 | 351 | FLYEYARRHPDYSVVLLLRLAK |
| albumin | F | 0.227 | 2.384 | 379 | 400 | PLVEEPQNLIKQNCELFEQLGE |
| albumin | G | 0.332 | 2.554 | 422 | 443 | TLVEVSRNLGKVGSKCCKHPEA |
| albumin | H | 0.191 | 2.735 | 439 | 460 | KHPEAKRMPCAEDYLSVVLNQL |
| albumin | I | 0.714 | 1.498 | 515 | 536 | TLSEKERQIKKQTALVELVKHK |
| prothrombin | A | 0.605 | 1.984 | 3 | 24 | TFLEEVRKGNLERECVEETCSY |
| prothrombin | B | 0.359 | 2.144 | 45 | 66 | TACETARTPRDKLAACLEGNCA |
| prothrombin | C | -0.141 | 3.263 | 170 | 191 | CVPDRGQQYQGRLAVTTHGLPC |
| prothrombin | D | 0.532 | 2.008 | 260 | 281 | LDEDSDRAIEGRTATSEYQTFF |
| prothrombin | E | 0.318 | 2.617 | 492 | 513 | VCKDSTRIRITDNMFCAGYKPD |
| prothrombin | F | 0.932 | 3.484 | 511 | 532 | KPDEGKRGDACEGDSGGPFVMK |
| apoC1 | A | 0.745 | 1.329 | 17 | 38 | TLEDKARELISRIKQSELSAKM |
| SAA1 | A | 0.345 | 1.64 | 9 | 30 | EAFDGARDMWRAYSDMREANYI |
| SAA1 | B | 0.495 | 1.684 | 13 | 34 | GARDMWRAYSDMREANYIGSDK |
| SAA1 | C | 0.491 | 1.761 | 60 | 81 | DARENIQRFFGHGAEDSLADQA |
| SAA1 | D | 0.409 | 3.645 | 81 | 102 | AANEWGRSGKDPNHFRPAGLPE |
| SAA2 | A | 0.345 | 1.64 | 9 | 30 | EAFDGARDMWRAYSDMREANYI |
| SAA2 | B | 0.495 | 1.684 | 13 | 34 | GARDMWRAYSDMREANYIGSDK |
| SAA2 | C | 0.65 | 1.64 | 60 | 81 | NARENIQRLTGRGAEDSLADQA |
| SAA4 | A | -0.259 | 1.956 | 6 | 27 | FFKEALQGVGDMGRAYWDIMIS |
| SAA4 | B | -0.045 | 2.128 | 13 | 34 | GVGDMGRAYWDIMISNHQNSNR |
| SAA4 | C | 0.009 | 2.355 | 40 | 61 | GNYDAAQRGPGGVWAAKLISRS |
| SAA4 | D | 1.141 | 3.634 | 89 | 110 | KAEEWGRSGKDPDRFRPDGLPK |

**Fig A**


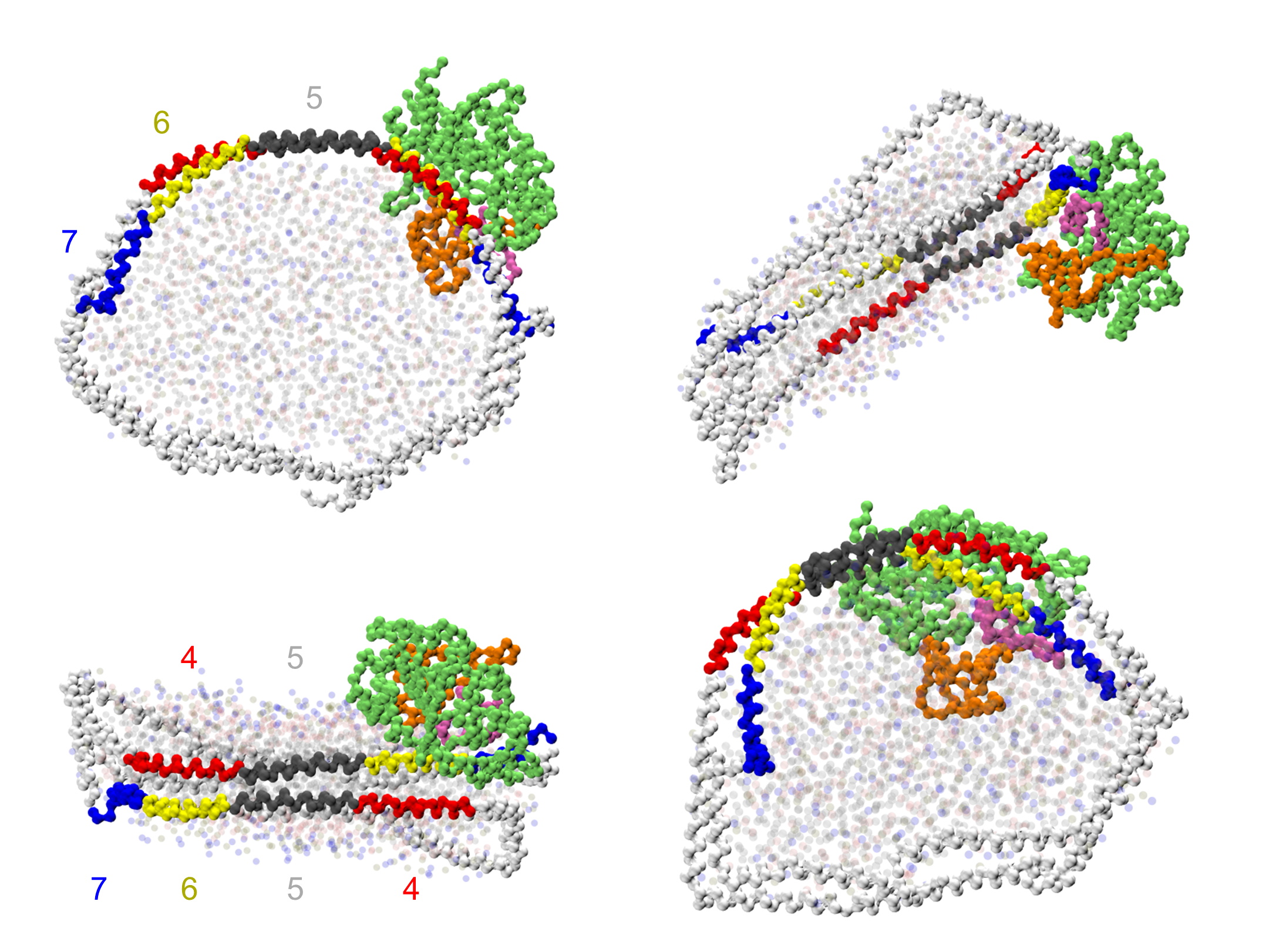


**Fig B**


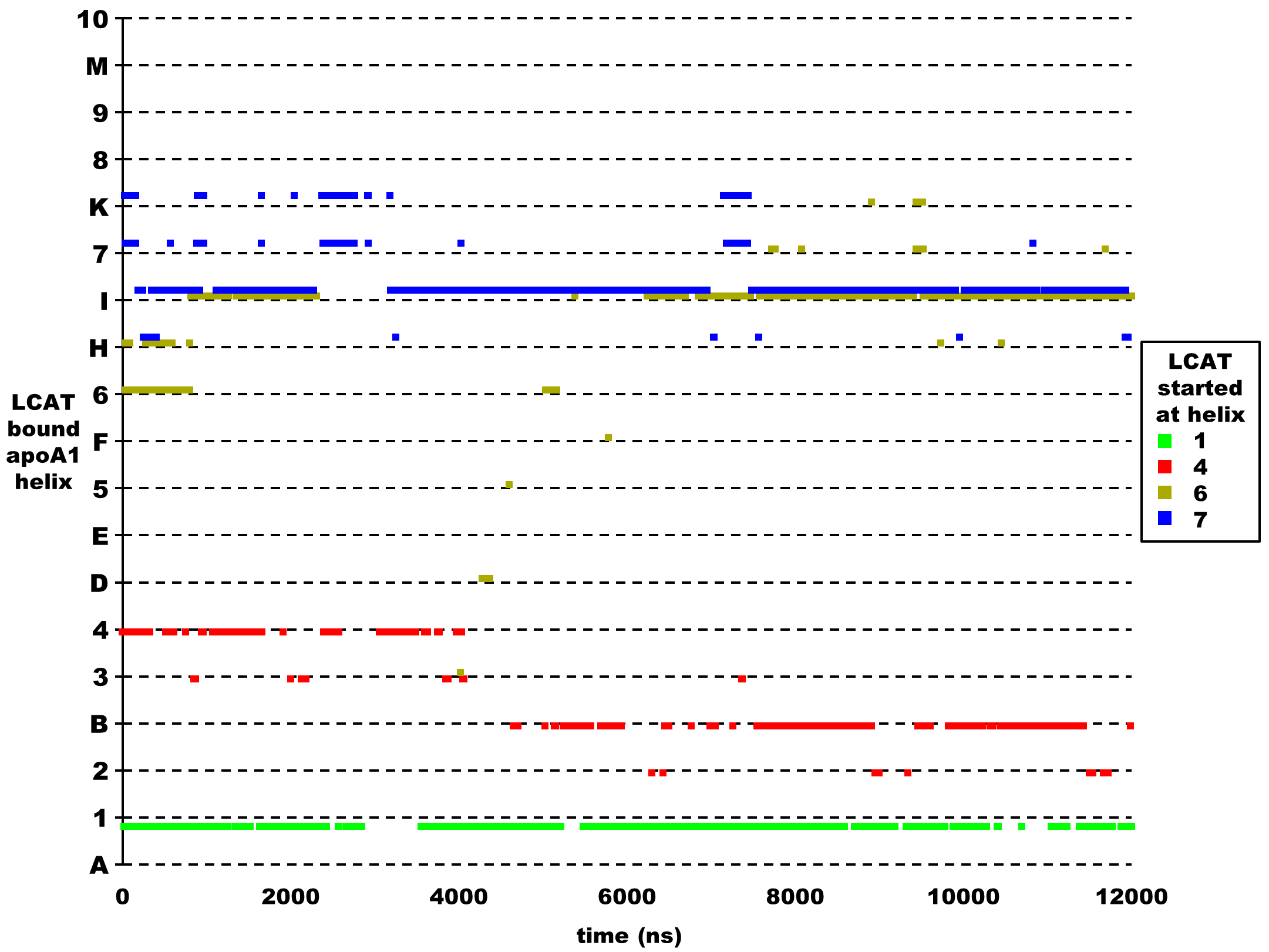


**Fig C**


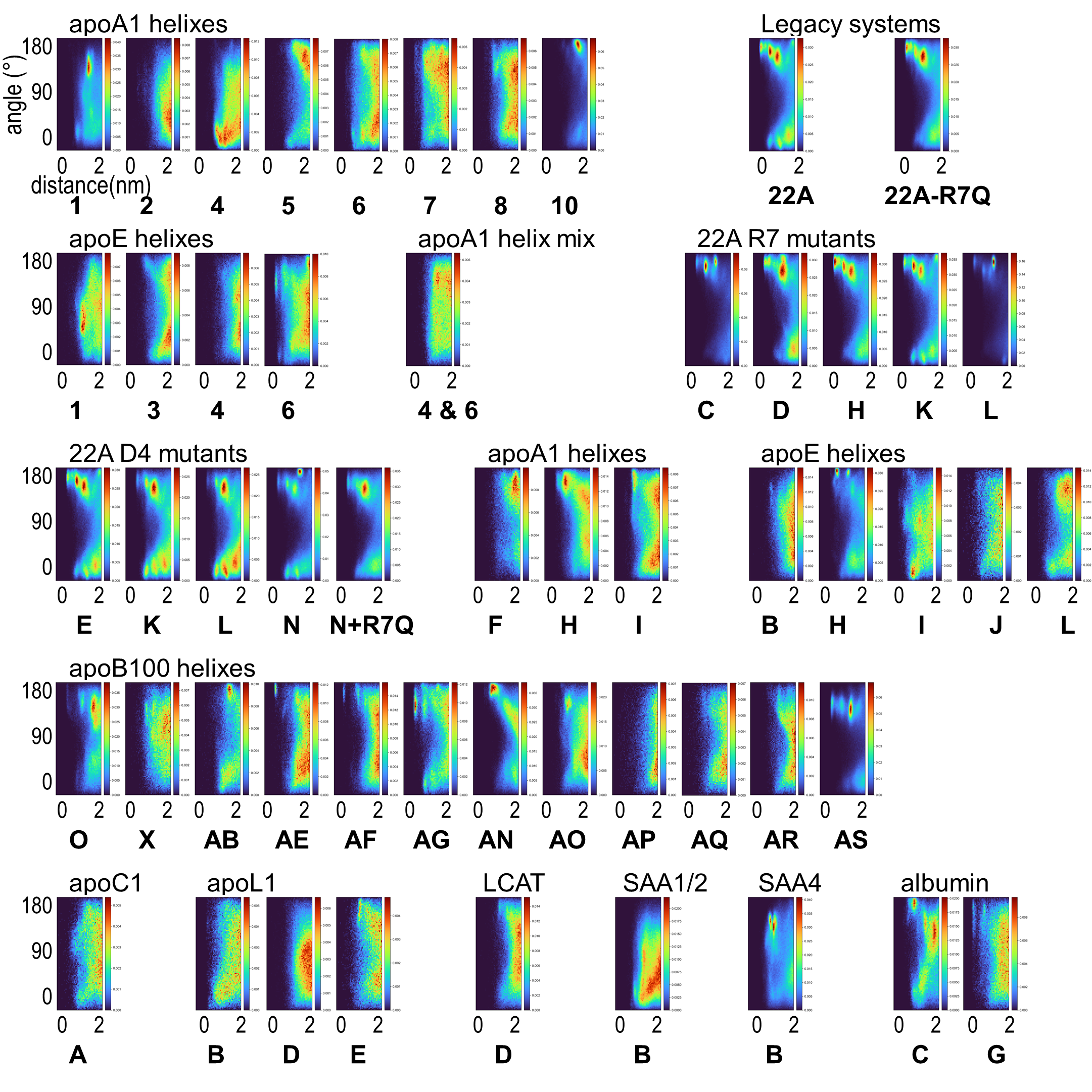


**Fig D**


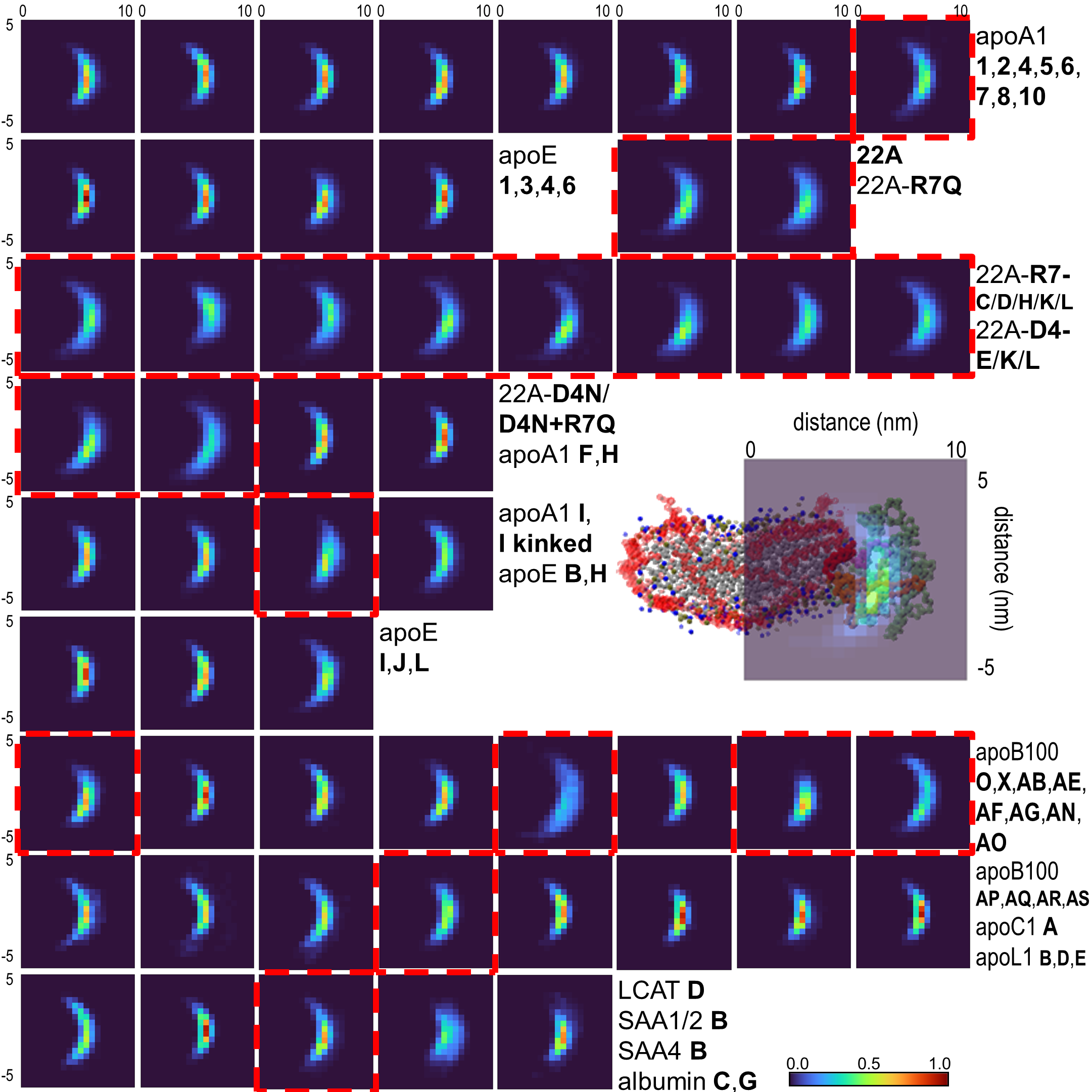


**Fig E**


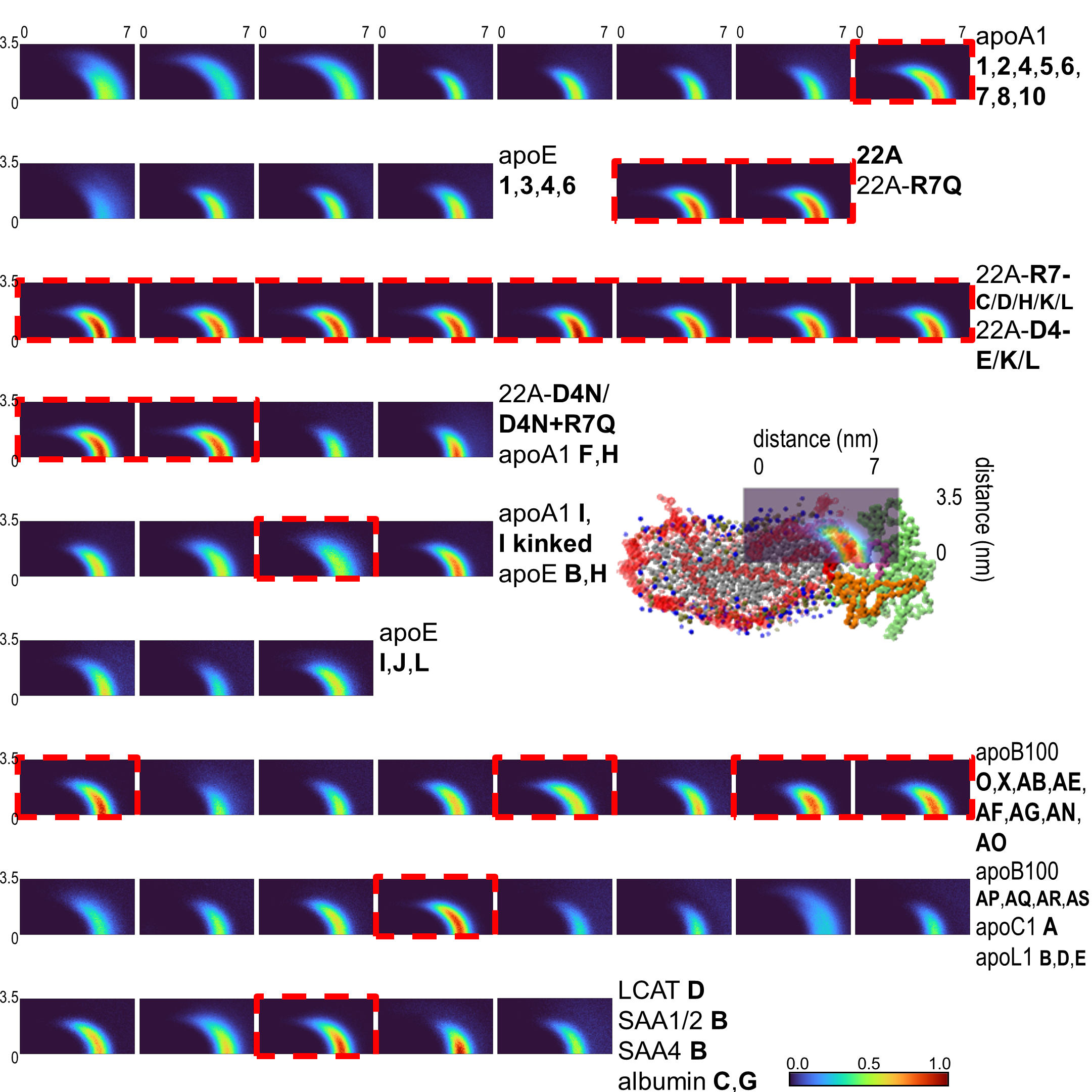


**Fig F**


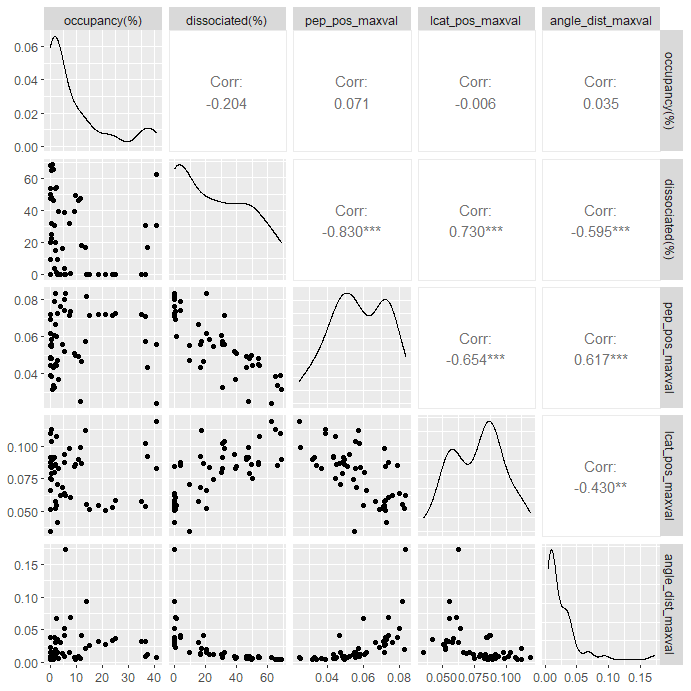


**Fig G**


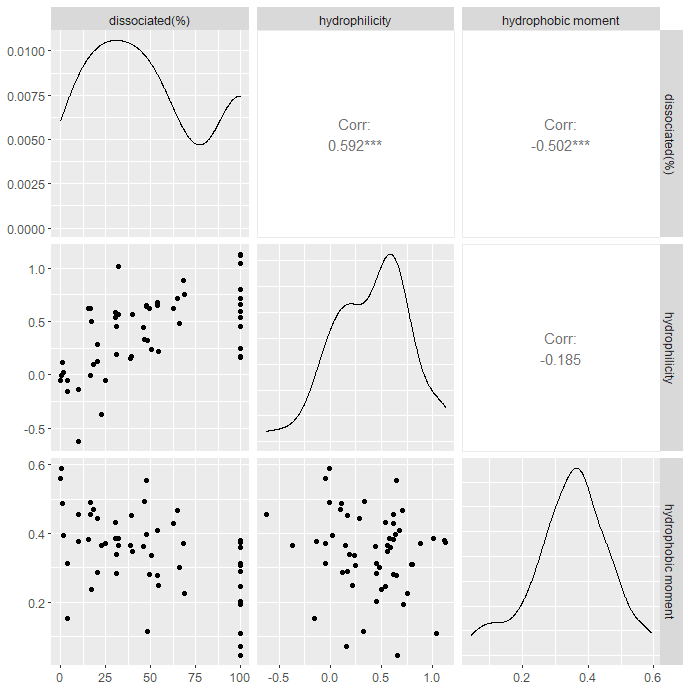

Supplement: S1 File — Table A. Peptide sequences in simulated systems. Table B. Hydrogen bonding results of atomistic systems. Peptide residues 4 and 7 and LCAT residues 53, 236 and 238 are bolded. Bonds with above 40% occupancy are shown. Table C. Occupancy results of 22A D4 mutation screen. Table D. All 22 residue long sequences with DE4-QR7 moieties in a variety of proteins. Fig A. LCAT binding pose from different perspectives on HDL particle. Snapshot of the last frame of helix 7 simulation. LCAT is coloured according to Fig 1 and apoA1 helixes according to Fig 4. Note the different position the peptide takes compared to Fig 1B. Fig B. The helix of apoA1 LCAT is bound to as a function of time. Simulations were started at helixes 1, 2, 4, 5, 6, 7, 8 and 10, but 2, 5, 8 and 10 were cancelled. As these helixes utilized a different binding orientation than 22 amino acid long peptides (Fig 1 vs Fig A) the occupancy condition was adjusted to any helix whose backbone bead distances between helix residue 2 and LCAT Q229 and helix residue 20 and LCAT L239 is within 1.2 nm. Fig C. Angle-distance profiles of CG systems with unnormalized colorbars. The apoA1 helix mix system’s bin counts are not comparable to other systems as it had 14 x 14 peptide pairs, unlike the other systems with 378 peptide pairs. Fig D. LCAT position density plots of CG systems. X-axis is distance from 0 to 10 nm perpendicular to nanodisc normal and Y-axis is distance from -5 to 5 nm parallel to nanodisc normal. A plane was fitted to all DMPC beads and LCAT’s position relative to it was determined with the same method as described in reference [1]. An illustrative superimposed image is included. The colorbar is normalized by the maximum bin count of apoE helix 1. Systems with less than 10% peptide dissociation are marked with a dashed red border. Fig E. Peptide position density plots of CG systems. X-axis is distance from 0 to 7 nm perpendicular to nanodisc normal and Y-axis is distance from 0 to 3.5 nm parallel to nanodi [file pcbi.1012137.s001.docx]
